# The differentiation of common species in a coral-reef fish assemblage for recreational scuba diving

**DOI:** 10.1186/s40064-016-3467-8

**Published:** 2016-10-10

**Authors:** Tsen-Chien Chen, Cheng-Tze Ho, Rong-Quen Jan

**Affiliations:** 1 Department of Leisure Management, Minghsin University of Science and Technology, No. 1, Xinxing Rd., Xinfeng Township, Hsinchu County, 304 Taiwan, ROC; 2 Fisheries Agency, Council of Agriculture, Executive Yuan, 6F., No. 100, Sec. 2, Heping W. Rd., Zhongzheng Dist., Taipei City, 100 Taiwan, ROC; 3 Biodiversity Research Center, Academia Sinica, No. 128, Sec. 2, Academia Rd., Nangang Dist., Taipei City, 115 Taiwan, ROC

**Keywords:** Coral reef, Ecological service, Dive tourism, Long-term data, Long-term monitoring

## Abstract

**Background:**

Recreational scuba diving is a popular activity of the coral reef tourism industry. In practice, local diving centers recommend interesting sites to help visiting divers make their plans. Fish are among the major attractions, but they need to be listed with care because the temporal occurrence of a fish species is difficult to predict. To address this issue, we propose methods to categorize each fish species based on its long-term occurrence and likelihood of being seen.

**Methods:**

We assume that there are K categories of occurrence of a fish assemblage and propose two methods [an arithmetic-mean method (AM) and a geometric-mean method (GM)] to define the range of species in each category.

**Results:**

Experiments based on long term datasets collected at three underwater stations (each having 51–53 surveys and totals of 262–284 fish species) on coral reefs in southern Taiwan showed that when K = 4 (rare, occasional, frequent and common categories), 11–14 species were concurrently assigned to the common category by AM for data sets based on surveys 10, 15, 20, 25, 30, 35, 40, 45, or 51–53 in contrast to the 18–26 species assigned as common by GM. If a similarity index of 0.7 (compared to the total pool of fish species) was the minimum threshold for diver satisfaction, then 20–25 surveys provide sufficient data for listing the common species at a given dive spot.

**Conclusions:**

Common fish species, are the most temporally stable, and thus are more appropriate for attracting divers. These can be effectively differentiated by either AM or GM with at least 25 surveys. We suggest regular updating of each fish’s category through periodic surveys to assure the accuracy of information at a particular dive spot.

## Background

Coral reefs are diverse and complex ecosystems that perform important ecological services and they have made significant contributions toward various human needs (Moberg and Folke 1999). Marine tourism flourishes around coral reefs where clear waters and hundreds of species of animals present a feast for eyes. Recreational scuba diving is considered an easy way to access wildlife. Diving and related services have developed into a fast growing sector of the tourism industry (Buzzacott 2008; Davis and Tisdell 1995; Hawkins et al. 2005; Stoeckl et al. 2010; Tongson and Dygico 2004). Fish are one of the tourist diver’s primary targets among sea creatures (Cater 2008; Stoeckl et al. 2010; Uyarra et al. 2009). So, providing information about fish-watching becomes essential when planning a dive.

Fish assemblages at coral reefs vary greatly in space and time, and are difficult to predict (Ault and Johnson 1998; Belmaker et al. 2005; Nanami and Nishihira 2003; Ormond and Roberts 1997; Sale et al. 1994; Syms and Jones 2000). Most species assemblages contain more rare species than common ones in terms of abundance (Jones et al. 2002; MacArthur and Wilson 1967; Fig. 2). When creating a recommended list of interesting fishes for divers, it is arguable whether a rare species should be included as its occurrence is possibly underlined by geographic range, sporadic abundance, and/or chance elements rather than by regional environmental factors (Jones et al. 2002). In contrast, a common fish species that lives steadily in the habitat is more likely to engender demand.

We are then left with the question of how to identify the common species in a fish assemblage. The decision cannot be made based on species abundance because the commonness of a species for diving purposes should be defined by the temporal stability of its occurrence rather than the number of individuals. To resolve this issue, we need a long-term dataset on species occurrence to use as a guideline to divide the species into different categories of occurrence. Moreover, guidelines must be clear enough to allow future cross-comparisons.

In this study, two methods based on long-term datasets were tested for categorizing fish species into multiple groups ranging from rare to common according to their temporal occurrence in the regional assemblage. Those species occurring commonly are recommended candidates for a list for fish-watching. This work is practical for evaluating wildlife watching resources and tourism.

## Methods

### Data collection

**Fig. 1:**
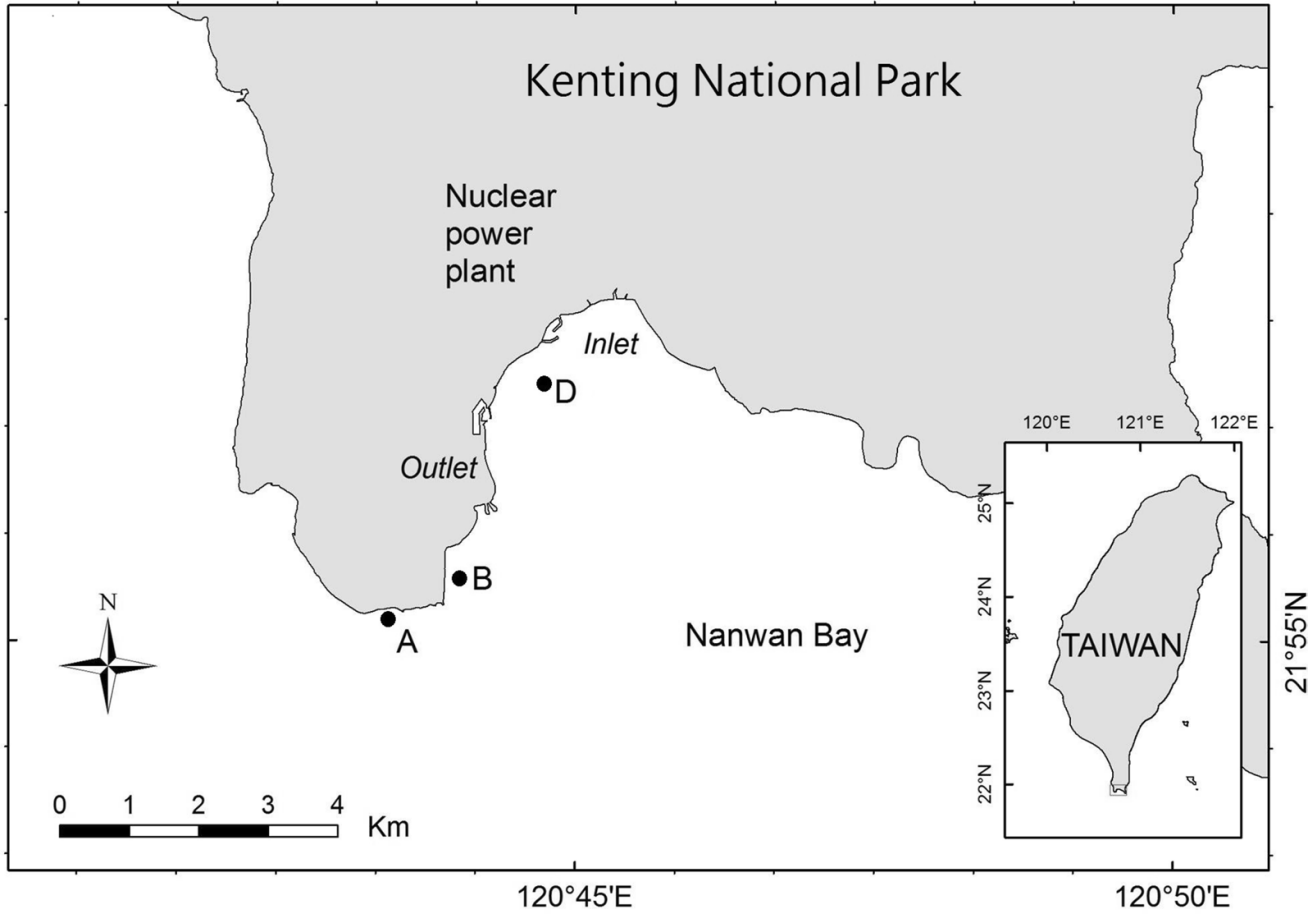
Map showing Nanwan Bay, Kenting National Park, and the study sites A, B and D, sub-tidal stations where monitoring was undertaken; Inlet, inlet of the water intake constructed by the nuclear power plant; Outlet, outlet of the water discharge canal

Field works were carried out in Nanwan Bay, Kenting National Park, at the southern tip of Taiwan (Fig. 1; Jan et al. 2001). Three sub-tidal stations (namely, Sts. A, B and D) on the coral reef area were assigned for long-term biomonitoring of thermal discharge from power plants. St. A, situated about 25 m from shore, is an angular block of 4 m (width) × 8 m (length) × 3 m (height) at a depth of 5–7 m. St. B, located 60 m from shore, is a limestone terrace of 20 m × 20 m × 6 m at a depth of 6–13 m. St. D, 500 m away from the shore, is composed of six adjacent rocks, the largest is 4 m × 3 m × 2 m at a depth of 10–12 m (Fig. 1). The underwater survey of fish assemblages was done using visual counts by scuba-diving ichthyologists who swam slowly and deliberately for 30 to 40 min to record fish species at each station. Taxonomic nomenclature for the fish has been carefully updated throughout the monitoring scheme to achieve taxonomic precision. The long-term monitoring of fish communities began in July of 1979 and continued along with associated monitoring programs until 1994, when the study was halted for financial reasons. Data collection was resumed in 1998–1999. The works were planned to be undertaken bimonthly. However, owing to weather conditions and some unanticipated situations, data from four to seven collections are available for each year.

### Defining categories

Assuming that N surveys have been conducted on a fish assemblage at constant time intervals, the minimum and maximum occurrence of a fish species in the assemblage would be 1 and N, respectively. Ideally, every species in the assemblage is assigned to one of K categories ranging from rare to very common according to the frequency of its occurrence.

**Fig. 2:**
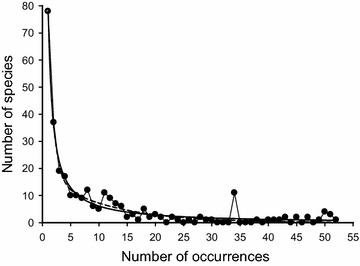
Number of fish species and their occurrences determined from 52 consecutive surveys undertaken by scuba divers at an underwater station in southern Taiwan (based on data collected from St. A). Among the 284 fish species observed, 78 occurred once, 37 twice, and 19 three times. The first three represented 47 % of the fauna. *Dashed line*: y = 180.1 × e^−1.1x^ + 17.7 × e^−0.1x^, R^2^ = 0.965; *solid line* y = − 0.39 + 63.5x^−1^ + 15.1x^−2^, R^2^ = 0.968

Two methods, the arithmetic-mean method (abbreviated AM) and the geometric-mean method (abbreviated GM), were used herein to define the range of each K category. Under AM, a common range of occurrence frequency X was shared by all categories, thus X = N/K. Species occurring in [(a − 1)X + 1] − aX (a = 1, 2,…,K) surveys were grouped into the ath category. In contrast, GM, which was inspired by the nonlinear relationship shown in Fig. 2, used the geometric mean (X = N^1/K^) to delineate the range of each category. Thus, species occurring in (X^a−1^) − X^a^ (a = 1) or (X^a−1^ +1) − X^a^ (a = 2, 3,…,K) surveys were classified into the ath category.

### Compositional similarity of common species

By definition, the frequency range of a category was dependent on the values of N and K. A total of 52 surveys were conducted at St. A (N = 52), 53 at St. B (N = 53), and 51 at St. D (N = 51). For each station, subsamples for surveys 10, 15, 20, 25, 30, 35, 40, and 45 were randomly selected from the data pool (Cao et al. 2003; Pos et al. 2014; Vellend et al. 2007), using statistical software package SPSS Version 17.0. Assuming K = 4 (rare, occasional, frequent, and common categories), then depending on the number of occurrences fish species were grouped into four different categories using the above methods.

Fish species attributed to the common category undertaken by different N were used for similarity comparison with that by the maximum N. The Jaccard’s similarity, an index for binary variables arrived at through calculating the quotient between the intersection and the union of pairwise compared variables among two objects, was employed (Kwak and Peterson 2007). For each station, random data sampling and similarity calculations were replicated three times.

## Results

**Fig. 3:**
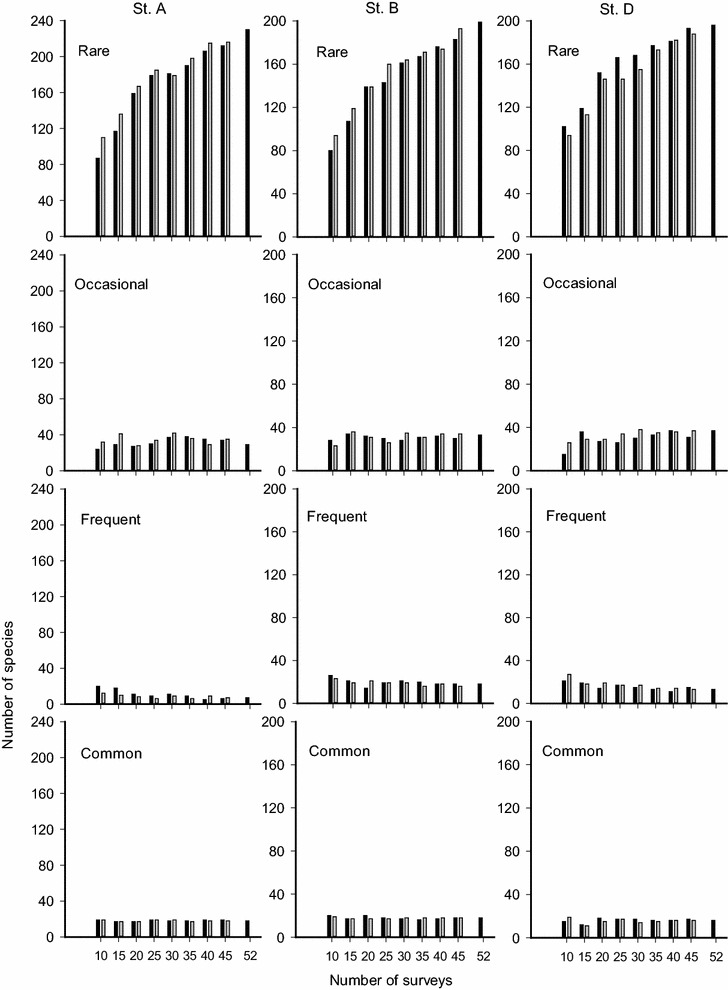
Numbers of rare, occasional, frequent, and common species categorized by the arithmetic mean method with K = 4 from different numbers of surveys in three coral reef fish assemblages (Sts. A, B and D). *Filled columns* denote data obtained from the species pool based on three replicates; *empty columns* represent the data obtained from one of them

**Fig. 4:**
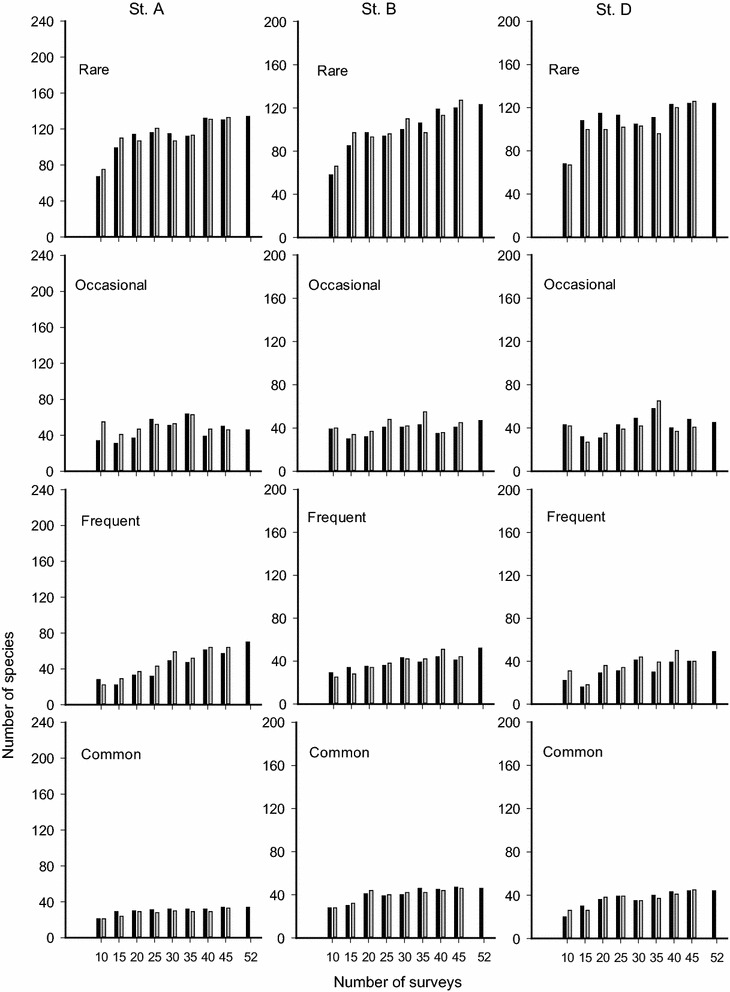
Numbers of rare, occasional, frequent, and common species categorized by geometric mean method with K = 4 from different numbers of surveys in three coral reef fish assemblages (Sts. A, B and D). Filled columns denote data obtained from the species pool based on three replicates; empty columns represent the data obtained from one of them

The long-term data set showed that a total of 284 fish species were recorded at St. A, 268 at St. B, and 262 at St. D. All three assemblages were dominated by rare (in the common sense) species (Fig. 2). Under both categorizing methods, rare species increased in number as N increased. Despite the changes in N, their role as the fauna’s major component did not change (Figs. 3, 4; only the data from one replicate from each station were shown because similar patterns occurred in all three replicates at each station).

### Arithmetic-mean method (AM)

**Fig. 5:**
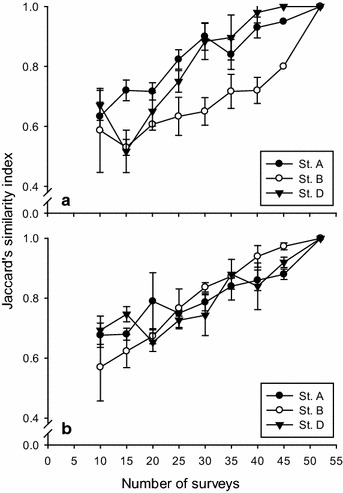
Jaccard’s similarity index (±1 SD) between common species obtained from 10 to 45 surveys (each with three replications) and from 52 surveys at three underwater stations (Sts. A, B and D). **a** Categorized by the arithmetic mean method; **b** categorized by the geometric mean method

The AM method calculated threshold numbers for occasional, frequent, and common categories were much lower than for rare species across Ns and stations (Fig. 3). At St. A, 17–19 common species were classified from data sets of varying Ns (Fig. 3). When N = 52, 18 common species were assigned. The coherent Jaccard’s similarity index value ranged from 0.58 to 0.95 between common species classified at N < 52 and N = 52. Changes of the index value followed a positive trend with the increase of surveys (Fig. 5a). There were 10 common species found concurrently at all Ns: five labrids, three pomacentrids, *Parupeneus multifasciatus*, and *Zanclus cornutus* (Table 1).

**Table 1:** Common species concurrently categorized based on surveys 10, 15, 20, 25, 30, 35, 40, 45, or 51–53 using an arithmetic mean method in three experiments at Sts. A, B and D

Station	Family	Species	Exp. 1	Exp. 2	Exp. 3
St. A	Labridae	*Gomphosus varius*	+	+	+
		*Halichoeres hortulanus*	+		+
		*Labroides dimidiatus*	+	+	+
		*Stethojulis bandanensis*			+
		*Thalassoma amblycephalum*	+	+	+
		*Thalassoma hardwickii*	+	+	+
		*Thalassoma lutescens*	+	+	+
	Mullidae	*Parupeneus multifasciatus*	+	+	+
	Pomacentridae	*Chromis margaritifer*		+	+
		*Neoglyphidodon nigroris*	+	+	+
		*Plectroglyphidodon dickii*	+	+	+
		*Pomacentrus philippinus*	+		
		*Stegastes fasciolatus*	+	+	+
	Tetraodontidae	*Canthigaster valentini*		+	+
	Zanclidae	*Zanclus cornutus*	+	+	+
St. B	Blenniidae	*Meiacanthus grammistes*			+
	Chaetodontidae	*Chaetodon kleinii*	+	+	+
	Labridae	*Labroides dimidiatus*	+	+	+
		*Macropharyngodon meleagris*		+	
		*Pteragogus enneacanthus*		+	+
		*Thalassoma amblycephalum*	+	+	
		*Thalassoma janseni*	+	+	+
		*Thalassoma lutescens*	+	+	+
	Mullidae	*Parupeneus multifasciatus*	+	+	
	Pomacentridae	*Chromis margaritifer*	+	+	+
		*Neoglyphidodon nigroris*	+		
		*Pomacentrus vaiuli*	+	+	+
		*Stegastes fasciolatus*	+	+	+
	Tetraodontidae	*Canthigaster valentini*	+	+	+
	Zanclidae	*Zanclus cornutus*	+	+	+
St. D	Chaetodontidae	*Chaetodon citrinellus*	+	+	+
		*Chaetodon kleinii*	+	+	+
	Labridae	*Halichoeres hortulanus*	+	+	+
		*Labroides dimidiatus*	+	+	+
		*Thalassoma amblycephalum*	+		+
		*Thalassoma lutescens*	+	+	+
	Pomacanthidae	*Centropyge vrolicki*	+	+	
	Pomacentridae	*Chromis margaritifer*	+	+	+
		*Chromis weberi*		+	
		*Dascyllus trimaculatus*	+	+	+
		*Pomacentrus vaiuli*	+	+	+
	Nemipteridae	*Scolopsis bilineatus*	+		
	Tetraodontidae	*Canthigaster valentini*	+	+	+
	Zanclidae	*Zanclus cornutus*	+	+	+

At St. B, 16–20 species were assigned to the common category (Fig. 2). When N = 53, 18 common species were assigned. The similarity index values ranged from 0.43 to 0.8 (Fig. 5a), with the lowest value (0.53) occurring when N = 15. Overall, there were 9 common fishes assigned concurrently at all Ns: three labrids, three pomacentrids, *Meiacanthus grammistes*, *Chaetodon kleinii*, *P*. *multifasciatus*, *Canthigaster valentini* and *Z*. *cornutus* (Table 1).

At St. D, 12–18 species were classified as common (Fig. 3). When N = 51, 16 common species were assigned. The similarity index values ranged from 0.62 to 1 (Fig. 5a). There were 10 common species assigned concurrently at all Ns: three labrids, three pomacentrids, two chaetodontids, *C. valentini*, and *Z*. *cornutus* (Table 1).

### Geometric-mean method (GM)

More species were grouped into the non-rare categories with the GM method than the AM method. That is, species numbers were more evenly distributed among categories with the GM method (Fig. 4). While the species numbers for the occasional and common categories varied only slightly, those in the frequent category followed a trend of increasing with increases in N (Fig. 4). GM modeling assigned more species to the common category because the range in the number of species in the common category using GM was wider than with AM. At St. A, 21–34 species were assigned to the common category (Fig. 4). When N = 52, 34 common species were assigned. The similarity index values ranged from 0.64 to 0.89 (Fig. 5b). There were 15 common species assigned concurrently across the varying Ns, including 9 common species assigned under AM plus *M. grammistes*, *Chromis margaritifer*, and four more labrids (Table 2).

**Table 2:** Common species concurrently categorized based on surveys 10, 15, 20, 25, 30, 35, 40, 45, or 51–53 separate surveys by using a geometric mean method in three experiments at Sts. A, B and D

Station	Family	Species	Exp. 1	Exp. 2	Exp. 3
St. A	Acanthuridae	*Ctenochaetus binotatus*	+		+
	Blenniidae	*Meiacanthus grammistes*	+	+	+
	Labridae	*Gomphosus varius*	+	+	+
		*Halichoeres hortulanus*	+	+	+
		*Labroides dimidiatus*	+	+	+
		*Macropharyngodon meleagris*	+	+	+
		*Pseudocheilinus hexataenia*	+	+	
		*Stethojulis bandanensis*	+	+	+
		*Thalassoma amblycephalum*	+	+	+
		*Thalassoma hardwickii*	+	+	+
		*Thalassoma janseni*	+	+	+
		*Thalassoma lutescens*	+		+
	Mullidae	*Parupeneus multifasciatus*	+	+	+
	Pomacentridae	*Chromis margaritifer*	+	+	+
		*Neoglyphidodon nigroris*	+	+	+
		*Plectroglyphidodon dickii*	+	+	+
		*Pomacentrus bankanensis*	+		
		*Pomacentrus philippinus*		+	
		*Stegastes fasciolatus*	+	+	
	Pseudochromidae	*Labracinus cyclophthalma*	+		+
	Tetraodontidae	*Canthigaster valentini*	+	+	+
	Zanclidae	*Zanclus cornutus*	+	+	+
St. B	Acanthuridae	*Ctenochaetus binotatus*	+	+	+
	Balistidae	*Sufflamen chrysopterum*	+		
	Blenniidae	*Meiacanthus grammistes*	+	+	+
	Chaetodontidae	*Chaetodon argentatus*	+		
		*Chaetodon kleinii*	+	+	+
	Labridae	*Coris gaimard*	+		
		*Gomphosus varius*	+		+
		*Halichoeres hortulanus*		+	+
		*Halichoeres nebulosus*	+		
		*Labroides dimidiatus*	+	+	+
		*Macropharyngodon meleagris*	+	+	
		*Pteragogus enneacanthus*	+	+	+
		*Stethojulis bandanensis*	+	+	+
		*Thalassoma amblycephalum*	+	+	+
		*Thalassoma hardwickii*	+	+	
		*Thalassoma janseni*	+	+	+
		*Thalassoma lutescens*	+	+	+
	Microdesmidae	*Ptereleotris evides*		+	+
	Mullidae	*Parupeneus multifasciatus*	+	+	+
	Pomacanthidae	*Centropyge vrolicki*	+	+	
	Pomacentridae	*Chromis margaritifer*	+	+	+
		*Neoglyphidodon nigroris*	+	+	
		*Plectroglyphidodon lacrymatus*		+	+
		*Pomacentrus bankanensis*		+	
		*Pomacentrus coelestis*			+
		*Pomacentrus philippinus*	+		+
		*Pomacentrus vaiuli*	+	+	+
		*Stegastes fasciolatus*	+	+	+
	Pseudochromidae	*Labracinus cyclophthalma*	+	+	
	Tetraodontidae	*Canthigaster valentini*	+	+	+
	Zanclidae	*Zanclus cornutus*	+	+	+
St. D	Acanthuridae	*Ctenochaetus binotatus*	+	+	+
	Caesionidae	*Pterocaesio digramma*			+
	Chaetodontidae	*Chaetodon auripes*	+		+
		*Chaetodon citrinellus*	+	+	+
		*Chaetodon kleinii*	+	+	+
	Labridae	*Bodianus mesothorax*	+		
		*Gomphosus varius*	+	+	+
		*Halichoeres hortulanus*	+	+	+
		*Labroides dimidiatus*	+	+	+
		*Pseudocheilinus hexataenia*	+	+	+
		*Thalassoma amblycephalum*	+	+	+
		*Thalassoma lutescens*	+	+	+
	Mullidae	*Parupeneus multifasciatus*	+	+	+
	Nemipteridae	*Scolopsis bilineatus*	+	+	+
	Pomacanthidae	*Centropyge vrolicki*	+	+	+
	Pomacentridae	*Chromis margaritifer*	+	+	+
		*Chromis weberi*	+	+	+
		*Dascyllus reticulatus*		+	
		*Dascyllus trimaculatus*	+		+
		*Pomacentrus vaiuli*	+	+	+
	Tetraodontidae	*Canthigaster valentini*	+	+	+
	Zanclidae	*Zanclus cornutus*	+	+	+

At St. B, 28–47 species were assigned to the common category (Fig. 4). When N = 53, 46 common species were assigned. The similarity index values ranged from 0.5 to 0.98 (Fig. 5b). There were 15 common species assigned concurrently across various Ns, including 9 common species assigned under AM plus *C. binotatus* and five more labrids (Table 2).

At St. D, 20–44 species were assigned to the common category. When N = 51, 44 common species were assigned. The similarity index values ranged from 0.64 to 0.94 (Fig. 5b). There were 17 common species assigned concurrently across various Ns, including 9 common species assigned under AM plus *Ctenochaetus binotatus*, *Centropyge vroliki*, *P. multifasciatus*, *Scolopsis bilineatus*, *Chromis weberi* and three more labrids (Table 2).

A total of 144 Jaccard’s index values (8 survey conditions × 3 replicates × 3 stations × 2 methods) were obtained. More than others, there were 42 data values falling between 0.7 and 0.79. With 20 or more surveys, index values ≧0.7 occurred across replicates, stations, and methods. The median value was 0.75, with 25 or more surveys. Herein, we suggested that 0.7 was the minimum threshold for discriminating common species using the concepts of statistical mode and median.

## Discussion

Exploring coral reefs through diving has increasingly become a popular marine recreational pursuit (Ong and Musa 2011). Underwater creatures that highlight scenic interests and the charms of a dive spot are the major tourism attraction (Cater 2008; Gray 1997; Nash and Chuk 2012; Sinha and Bushell 2002). In this study, we raise the concept and propose two methods of delineating common species to highlight the uniqueness of fish fauna at dive locations.

Commonness is often used in ecology to explain the population status of a species (Gaston 1994, 2010). A species prevailing over a wide geographical range and having broad habitat specificity, large local abundance, and frequent habitat occupation can be considered a common species (Rey Benayas et al. 1999). Commonness is normally closely related to abundance, referring to the frequency with which a species is found in controlled areas (Hu et al. 2012; Sizling et al. 2009; Zacharias and Brandes 1990). In this study, the definition of commonness was adjusted from spatial abundance to temporal presence in order to meet the demands arising from visits by different divers. The “star” species used to lure tourists to many destinations are good examples. Longfin batfish on the steel-frame reefs of Green Island, Taiwan (Yang et al. 2011), hammerhead sharks at Layang Layang Island, Malaysia (Musa et al. 2006), Nassau groupers at the Turks and Caicos Islands and Bahamas (Rudd and Tupper 2002), and pygmy-seahorses in Kimbe Bay, Papua New Guinea (Cater 2008) are some of the star species often showcased in diving magazines, guide books, promotional videos, and websites. Their appearance and behavior can trigger tourist diver interest. However, there should also be a focus on their reliability of occurrence, a factor essential for guaranteeing satisfactory encounters on commercial dive trips.

Applications of the two methods based on long-term data sets demonstrate that the “commonness” of a fish species will not vary when data sets reach a certain size. These two methods are therefore both effective, and easy to use. Moreover, prior to accurate predictions of fish occurrences being available, these methods might allow for cross-examination for temporal occurrences of fish species in different assemblages.

Using either an AM or a GM method, each fish species is assigned a category of occurrence. However, since by design the results of both methods are dependent on the number of surveys, the occurrence attribute of a species can change with changes in N. the similarity index plots (Fig. 5) demonstrate that similarity values are low at small Ns and get higher with a positive link to the increase of N. Accumulating long-term monitoring datasets can be expensive in time and funding. Indeed, our results indicate that if a similarity index value of 0.7 is assumed to represent the minimum degree of demand, then the data that is accumulated from 20 to 25 surveys would be required to list a site’s common species. However, because environments are ever-changing, and increasingly more so in recent years (Cheung et al. 2009; Haward et al. 2013; Graham et al. 2014; Lavides et al. 2016), it is helpful to regularly update a fish’s category by adding new surveys covering various seasons to document current fish assemblages to assure the accuracy of the wildlife information available for a dive spot.

## Conclusions

Despite the temporal variability of fish species abundance, the need for lists of fish species which are interesting to divers is important for the tourist industry. Accurate predictions of temporal species composition within fish assemblages have been very difficult to make because the occurrence of a species is governed by variables including recruitment, biological associations, food and habitat specificity, and predator–prey relationships. The present study reduces temporal variability by placing fish into species occurrence categories, and shows that data based on 20–25 surveys is needed when using either an AM or a GM method (assuming K = 4) to identify the common species at dive spots. Common fish species are the most temporally stable, and thus are more appropriate for attracting divers.
